# The Burden of Gastrointestinal Complaints in Kidney Transplant Recipients Using Tacrolimus With and Without Mycophenolate Mofetil: A Randomized Controlled Study

**DOI:** 10.3389/fneph.2022.933954

**Published:** 2022-07-19

**Authors:** Zainab Al Fatly, Michiel GH Betjes, Judith van Gestel, Marieken Verschragen, Annelies E. de Weerd

**Affiliations:** Department of Internal Medicine, Erasmus Medical Center Transplant Institute, University Medical Center Rotterdam, Rotterdam, Netherlands

**Keywords:** kidney transplantation, mycophenolate mofetil, tacrolimus, immunosuppression, gastrointestinal complaints, GSRS questionnaire, diarrhea

## Abstract

**Background:**

Tacrolimus (TAC) combined with mycophenolate mofetil (MMF) is the immunosuppressive regimen in the majority of solid organ transplant recipients. Gastrointestinal complaints are frequent, which is considered predominantly a side effect of MMF. However, systematic research in this field is lacking. The aim of this study is to systematically investigate the burden of gastrointestinal complaints in TAC-treated kidney transplant recipients with and without MMF.

**Methods:**

In a single-center, open-label, randomized controlled trial, low immunological risk recipients were randomized to either TAC and MMF or to TAC monotherapy from 6 months after kidney transplantation onwards [NTR4672],. They filled in the Gastrointestinal Symptom Rating Scale questionnaire, which covers five dimensions (abdominal pain, reflux, indigestion, constipation, and diarrhea), 6, 12, and 15 months after transplantation.

**Results:**

Seventy-nine recipients were randomized and 72 completed all questionnaires (34 TACmono and 38 TAC/MMF). At baseline, the mean age was 59 years with 72% male, mean BMI 28 kg/m^2^, eGFR 55 ml/min/1.73m2, mean daily dose MMF 1200 mg and TAC 5.8 mg, with trough levels of 2.1 mg/L and 7.4 ug/L. Six months after transplantation, 75% of recipients reported troublesome symptoms (score ≥3). Diarrhea was the most troublesome (mean 3.3) and discontinuing MMF significantly reduced it (mean Δ score between month 6 and 15 TAC/MMF -0.9 vs. TACmono -1.8, p=0.03). In recipients with troublesome symptoms, abdominal pain (2.7 to 1.8, p=0.003), indigestion (2.8 to 2.3, p=0.012), and reflux (2.9 to 1.7, p=0.007) significantly decreased over time, independent of MMF use.

**Conclusion:**

The majority of kidney transplant recipients with TAC and MMF experienced troublesome gastrointestinal symptoms 6 months after transplantation. While constipation remained troublesome, indigestion, abdominal pain, and reflux improved over time by month 15. Diarrhea only improved after discontinuing MMF.

## Introduction

Gastrointestinal symptoms are common in kidney transplant recipients. Prevalence rates vary from 20 up to 92% of mild to severe gastrointestinal symptoms (1–3). Though frequent, gastrointestinal symptoms are often underestimated by physicians (4).

Immunosuppressive drugs and infections are well-known causes of gastrointestinal symptoms (5). Common infections are CMV, Helicobacter pylori, Candida, and chronic norovirus (6). Immunosuppressive drugs can result in gastrointestinal symptoms *via* direct side effects of the drugs and *via* its immunosuppressive mechanism leading to a higher susceptibility for (opportunistic) infections (7).

Gastrointestinal symptoms are common in kidney transplant recipients treated with mycophenolate mofetil (MMF) (5, 7). In fact, gastrointestinal symptoms in recipients on MMF regularly lead to discontinuation of MMF (2). Clinical studies on gastrointestinal symptoms and MMF often study conversion to enteric-coated MMF or have been performed during registration studies with azathioprine as control treatment next to cyclosporine (2, 8–10). Nowadays, standard immunosuppressive treatment after kidney transplantation mostly consists of tacrolimus (TAC), MMF, and (at least temporarily) corticosteroids. TAC is also associated with diarrhea, however since TAC is mostly combined with MMF, its contribution to gastrointestinal symptoms is difficult to assess. Randomized studies on gastrointestinal symptoms with TAC in the presence and absence of MMF are lacking. However, the impact of gastrointestinal symptoms in kidney transplant recipients is substantial as it may lead to diminished quality of life (1), non-adherence (7, 11), and even graft loss (2). Furthermore, since gastrointestinal symptoms are often underreported, the burden of gastrointestinal symptoms may be overlooked when not systematically investigated (1, 3).

We have performed a randomized controlled study in kidney transplant recipients with low immunosuppressive risk who either continued dual therapy with TAC and MMF or discontinued MMF and were treated with TAC monotherapy. The randomized design offers a chance to study and quantify the individual contribution of MMF as opposed to TAC combined with MMF. Furthermore, the course over time of gastrointestinal symptoms in kidney transplant recipients can be explored.

## Materials and Methods

### Study Design

This cohort study was part of a randomized controlled trial, which was an investigator-driven, open-label, single center pilot study. This trial was approved by the institutional review board of the Erasmus Medical Center (MEC-2014-318) and registered in the Netherlands Trial Register [NTR4672, www.trialregister.nl]. Randomization was computer-generated, in a 1:1 ratio to either continue TAC and MMF or to halve MMF at month 6 and discontinue it at month 9.

**Figure 1:**
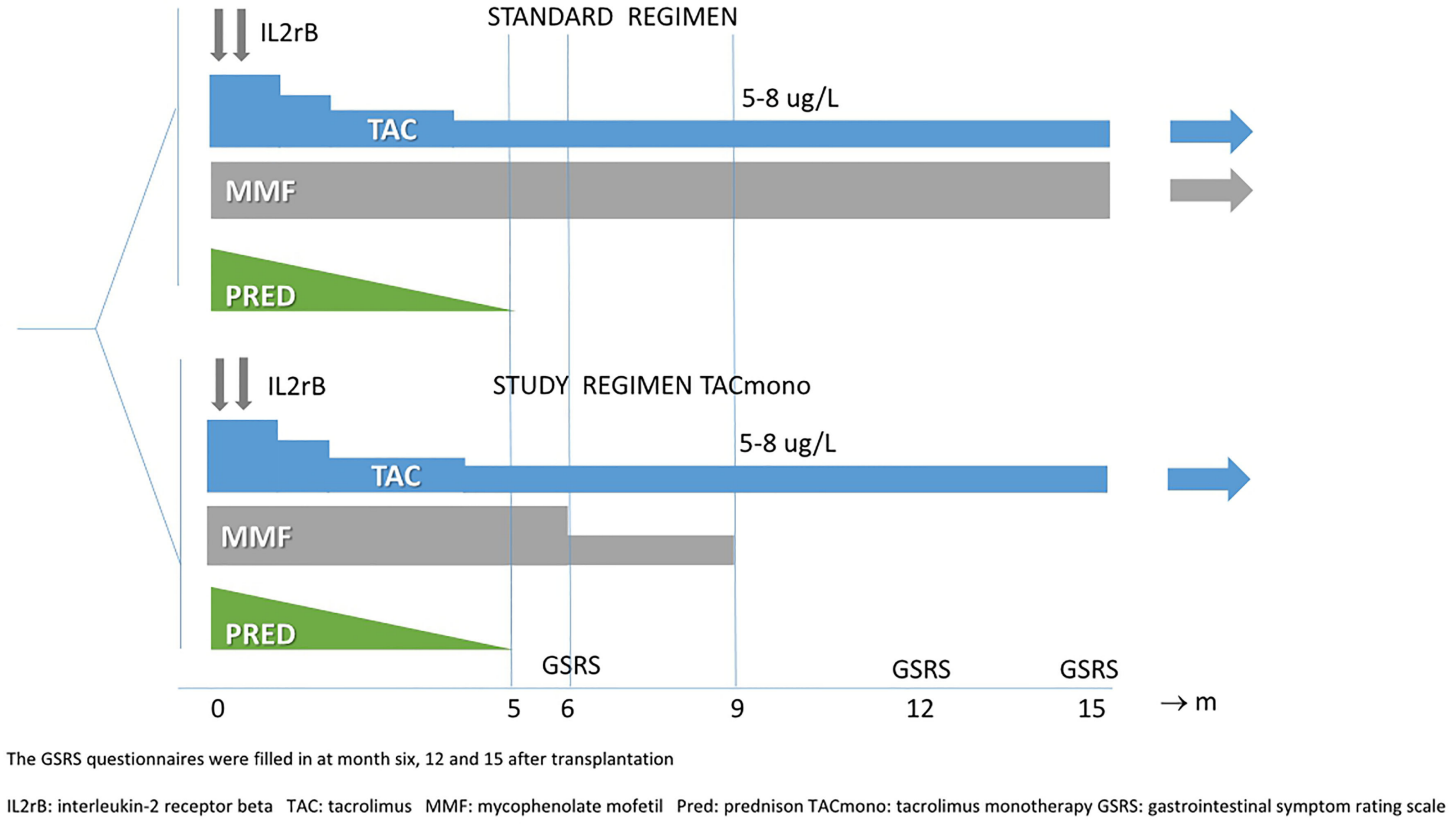
Treatment arms.

Target trough levels were 5-8 ug/L for TAC in both arms and 1.5-3 mg/L for MMF in the standard arm (see **Figure 1** for immunosuppressive regimens and trough levels). Study visits with the research nurse took place at 6, 12, and 15 months after transplantation in the outpatient clinic. The aims of this study were to systematically investigate gastrointestinal symptoms in immunologically low-risk kidney transplant recipients and in particular the impact of MMF on top of TAC.

### Patients

Between 2014 and 2018, recipients were included at time of kidney transplantation, after obtaining written informed consent. Inclusion criteria were age above 18, ABO-blood group compatible kidney transplantation, HLA mismatches of three or lower, and peak Panel Reactive Antibodies (PRA) of ≤4%. The main exclusion criterion was an immunological disease requiring additional immunosuppression (on top of tacrolimus). After a run-in period of 6 months, the included recipients were randomized if eGFR was >30 ml/min/1.73 m2 (CKD-EPI), proteinuria <0.5 mg/mmol, if there was freedom of biopsy-proven acute rejection (BPAR) from months 3 onwards, and if no depleting therapy had been administered. The full inclusion, exclusion, and randomization criteria are depicted in **Supplemental Table S1**. Prednisone was discontinued at month 5.

### GSRS Questionnaires

Recipients filled in the Gastrointestinal Symptom Rating Scale (GSRS) questionnaire (©AstraZeneca,1995. GSRS – The Netherlands-Dutch). This questionnaire is a self-report instrument and contains 15 questions on gastrointestinal symptoms. It makes use of a seven-point graded Likert scale, where 1 represents the absence of symptoms and 7 represents very troublesome symptoms. The GSRS questionnaire covers five gastrointestinal dimensions (covering abdominal pain, reflux, diarrhea, indigestion, and constipation symptoms). Patients filled in the GSRS questionnaires at 6, 12, and15 months at the outpatient clinic. In case of low literacy skills, the research nurse assisted in answering the questionnaires.

### Outcomes

Gastrointestinal symptoms were assessed per symptom, per dimension, and per category of upper and lower gastrointestinal symptoms. Gastrointestinal symptoms are present with a score of 2 and higher, and a score of ≥3 is defined as a troublesome symptom. If at least one question in a domain has a score of ≥3, then the domain qualifies as troublesome. So, a troublesome dimension could have a mean score lower than 3, as its score is the mean of three to five questions.

### Statistical Analysis

A per-protocol analysis was performed, comparing TAC monotherapy to TAC combined with MMF. A three-factor analysis was performed with month 6, 12, and 15. Means per dimension and per category (upper and lower GI symptoms) were calculated in Microsoft Excel (version 15.25). Independent samples t-test was used to compare differences between treatment groups using IBM SPSS statistics (version 25). Repeated measures ANOVA test was used to measure the interaction of MMF with gastrointestinal symptoms over time. Regression analysis was performed using IBM SPSS statistics (version 25). The variable antibiotic use was defined as at least 3 consecutive days of antibiotic use in the 3 months prior to filling in the questionnaire. If the samples were of unequal size and not normally distributed, a Mann Whitney U-test was performed. Prism 6 (GraphPad Prism, version 5.01) was used to make figures. A p-value of <0.05 was considered statistically significant.

## Results

Between 2014 and 2018, 121 kidney transplant recipients were included in the main study. Two of them had terminated the study before randomization due to discontinuation of MMF because of diarrhea. This discontinuation was done at the discretion of their treating physician and their complaints resolved subsequently.

**Figure 2:**
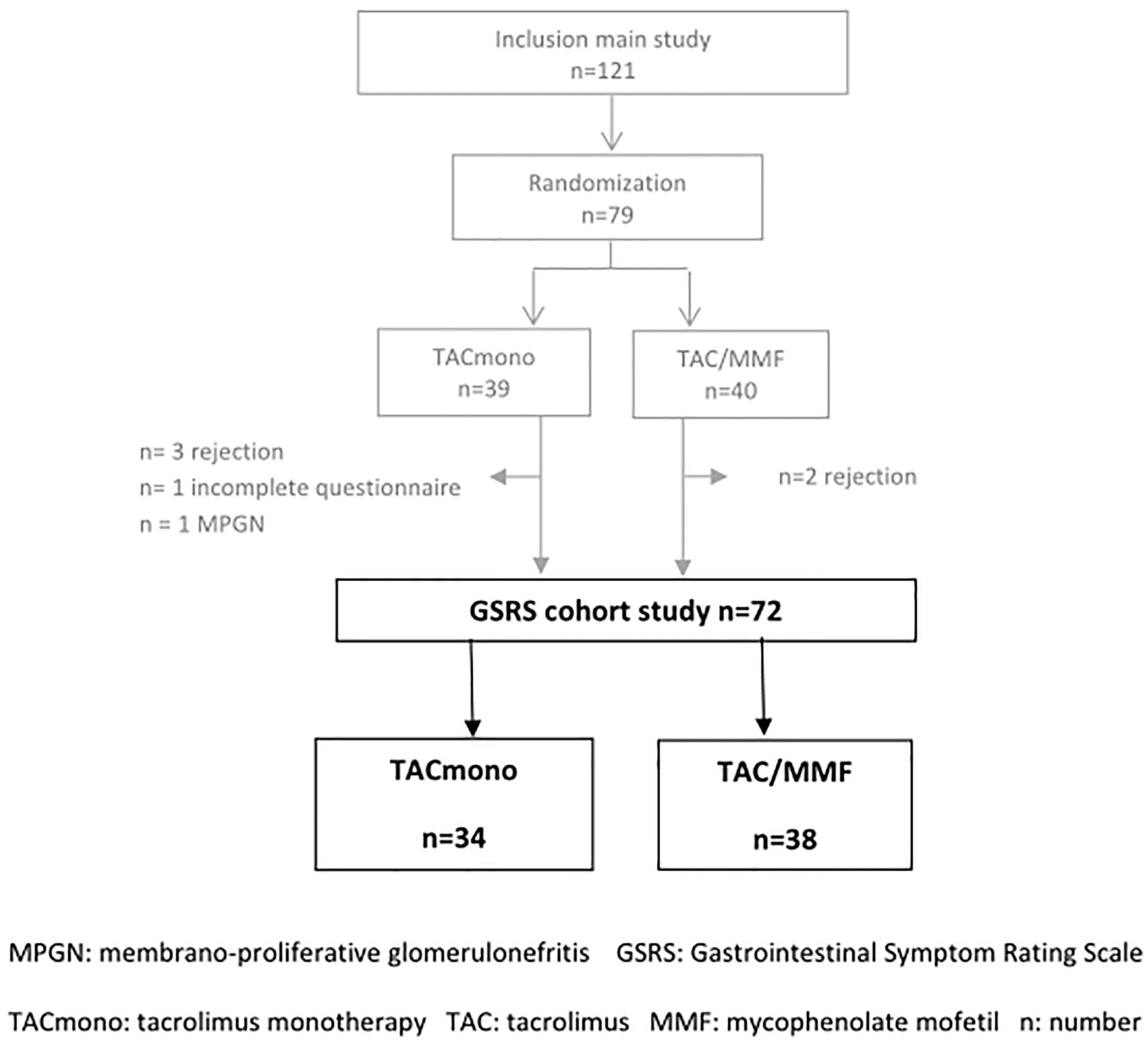
Patient enrollment.

After the run-in period of 6 months, 79 recipients met the randomization criteria and were randomized to either TAC/MMF (n=41) or to TACmono (n=38). Seventy-two recipients completed all GSRS questionnaires while still in the treatment arm and were included in the current GSRS study. This resulted in a study cohort of 34 TACmono and 38 TAC/MMF recipients. Of the seven excluded recipients, two had incomplete questionnaires and five did not complete the main study due to rejection (**Figure 2**).

### Baseline Characteristics

Baseline characteristics did not differ between groups (**Table 1**). Mean age was 59 years. The majority of recipients, 52 out of 72 (72%), were male. Self-reported ethnicity was 63% Caucasian, 17% Asian, 13% African, and 7% Middle Eastern. Fifty-six percent of the recipients had a low level of education, according to the definition of the Dutch central agency of statistics (12). Most transplants were from living donors (61%). Mean eGFR at 6 months after transplantation was 56.3 ml/min/1.73 m2. The trough levels of TAC and MMF did not differ between the groups and were within target range (mean 7.4 vs. 7.2 ug/L and 2.2 vs. 1.8 mg/L, respectively, in TAC vs. TAC/MMF) at 6 months after transplantation. Median dose of TAC was 6.0 vs. 5.0 mg daily in TAC vs. TAC/MMF and median dose of MMF was 1000 mg daily in both groups.

**Table 1:** Baseline characteristics of recipients 6 months after kidney transplantation.

	TAC-mono(n = 34)	TAC/MMF(n = 38)
Age, median (range in years)	63 (37-71)	61 (29-80)
Sex, n male (%)	25 (74%)	27 (71%)
BMI, median (range in kg/m^2^)	28 (21-36)	27 (19-34)
Transplant type, n living donor transplant (%)	23 (68%)	21 (55%)
Proteinuria protein/creatinine ratio, median (range in mg/mmol)	12.1 (0-34.5)	11.9 (6.8-46.6)
CKD-EPI eGFR, median (range in ml/min/1,73 m^2^)	59 (32 – 105)	55 (30 – 84)
TAC trough level, median in ug/l (IQR)	7.4 (2.8)	7.2 (2.8)
MMF trough level, median in mg/l (IQR)	2.2 (1.5)	1.8 (1.4)
Daily dose TAC, median in mg (IQR)	6.0 (5.4)	5.0 (4)
Daily dose MMF, median in g (IQR)	1.0 (0.9)	1.0 (0.5)

TACmono, tacrolimus monotherapy; n, number; BMI, body mass index; eGFR, estimated gromerular filtration rate; TAC, Tacrolimus; MMF, Mycophenolate mofetil; IQR, Interquartile range.

### Gastrointestinal Symptoms in Kidney Transplant Recipients with TAC/MMF

**Figure 3:**
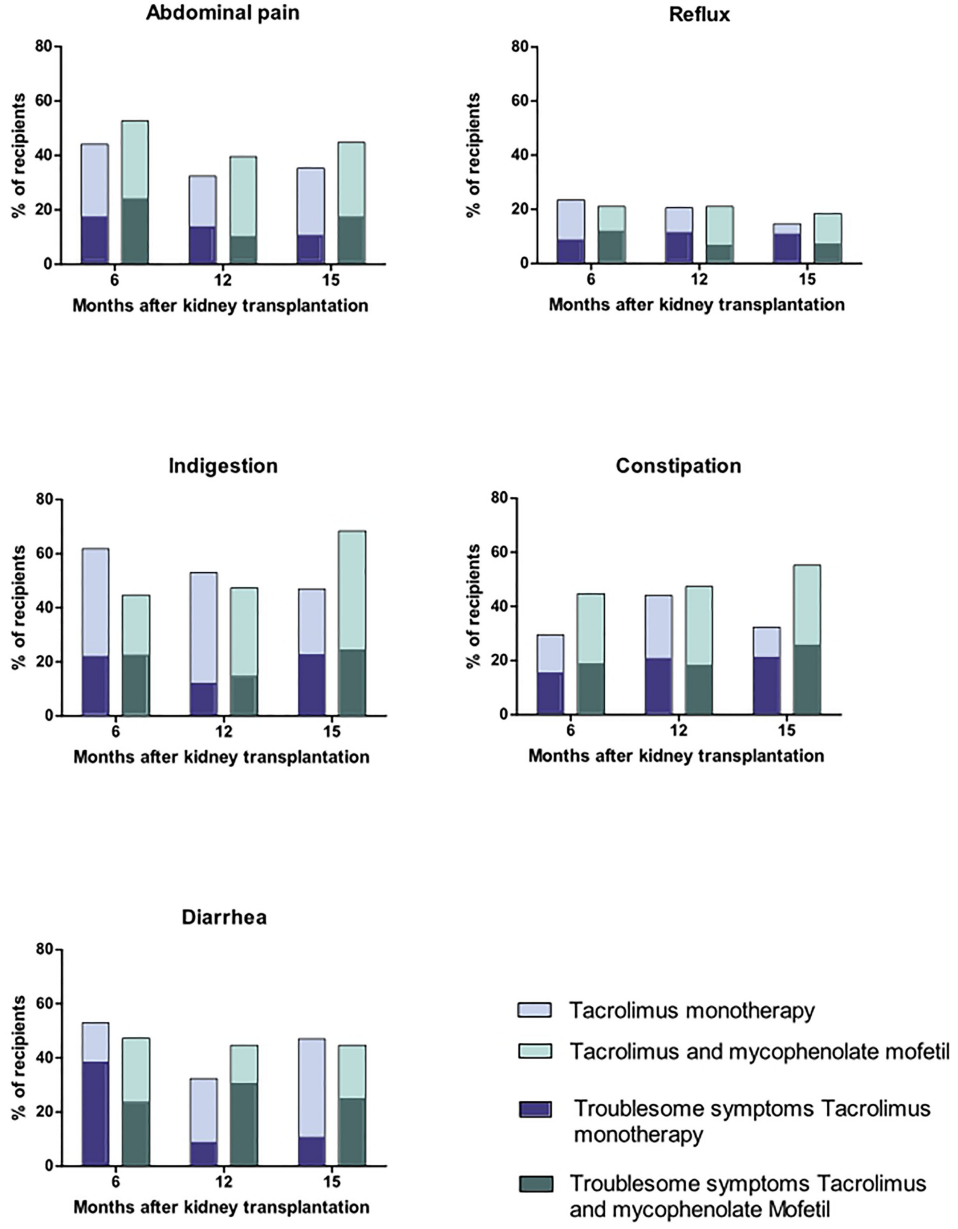
Gastrointestinal symptoms in stable, immunologically low-risk kidney transplant recipients with tacrolimus and mycophenolate mofetile versus tacrolimus monotherapy.

Six months after kidney transplantation, 90% of the recipients (65/72) reported gastrointestinal symptoms (score ≥2), and 75% of the recipients (54/72) reported troublesome gastrointestinal symptoms (score ≥3, **Figure 3**).

The most reported symptoms at 6 months were in the dimension indigestion (**Figure 3**).

The dimension diarrhea was the dimension with the highest mean score (2.1, **Figure 3** and **Table 2**). The dimension diarrhea consisted of three questions on the GSRS questionnaire, which were:

- Have you been bothered by DIARRHEA during the past week?
- Have you been bothered by LOOSE STOOLS during the past week?
- Have you been bothered by an URGENT NEED TO HAVE A BOWEL MOVEMENT during the past week?

**Table 2:** Gastrointestinal symptoms in five dimensions, TACmono vs. TAC/MMF.

Time	Dimension	TAC-monomean (SD)	TAC/MMFmean (SD)	P-value*
**Month 6**	**Abdominal pain**	1.7 (1.05)	1.9(1.03)	
	**Reflux**	1.3 (0.64)	1.4(0.95)	
	**Indigestion**	2.0 (1.09)	1.8(1.10)	
	**Constipation**	1.5 (0.96)	1.7 (1.08)	
	**Diarrhea**	2.4 (1.69)	1.8(1.04)	
**Month 12**	**Abdominal pain**	1.5 (0.83)	1.6 (0.86)	0.75
	**Reflux**	1.4 (0.93)	1.3(0.61)	0.48
	**Indigestion**	1.7 (0.84)	1.7(0.84)	0.86
	**Constipation**	1.8 (1.05)	1.8(1.22)	0.69
	**Diarrhea**	1.5 (0.96)	1.9(1.12)	0.17
**Month 15**	**Abdominal pain**	1.5 (0.86)	1.7(0.84)	0.55
	**Reflux**	1.3 (0.76)	1.3(0.88)	0.88
	**Indigestion**	1.7 (0.93)	2.0(0.97)	0.20
	**Constipation**	1.6 (0.96)	2.1(1.36)	0.10
	**Diarrhea**	1.7 (1.02)	1.8(1.18)	0.60
**Change over time from 6 until 15 months after transplantation.**				
		**Δ score**	**Δ score**	***p-value* ^#^**
	**Abdominal pain**	- 0,2	- 0.2	0.87
	**Reflux**	- 0,0	- 0.1	0.61
	**Indigestion**	- 0,2	+ 0.3	0.12
	**Constipation**	+ 0,1	+ 0.3	0.23
	**Diarrhea**	- 0,7	0.0	0.04

*Independent samples t-test # Repeated measures ANOVA test SD, standard deviation.

TAC, Tacrolimus; MMF, Mycophenolate mofetil.

Mean scores for the other dimensions were 1.8 for abdominal pain, 1.4 for reflux, 1.9 for indigestion, and 1.6 for constipation (**Table 2**).

In the standard immunosuppressive regimen TAC/MMF, the course of gastrointestinal complaints revealed a more or less stable pattern with no significant changes between 6, 12, and 15 months after transplantation. Mean scores at 6, 12, and 15 months were for abdominal pain 1.9, 1.6, and 1.7; for reflux 1.4, 1.3, and 1.3; for indigestion 1.8, 1.7, and 2.0; for constipation 1.7, 1.8, and 2.0; and for diarrhea 1.8, 1.9, and 1.8, all non-significant (**Table 2**).

### The Impact of MMF on Gastrointestinal Symptoms

Discontinuing MMF led to a significant improvement of diarrhea symptoms between 6 and both 12 and 15 months after transplantation, that is 3 and 6 months after discontinuation of MMF (p=0.04). In TACmono, diarrhea symptoms decreased from a mean score of 2.4 at month 6 to 1.7 at month 15, whereas in TAC/MMF diarrhea remained stable with mean scores of 1.8 at 6 and at 15 months (**Table 2**). The comparison of upper and lower gastrointestinal symptoms revealed no significant differences between TACmono and TAC/MMF (**Supplemental Table S2**).

Furthermore, diarrhea complaints diminished when MMF dose was lowered. These complaints increased when the dose remained unchanged or was increased at month 12 compared to month 6 after kidney transplantation. The mean diarrhea score increased from 1.4 to 1.9 when the MMF dose was increased and from 1.4 to 1.7 when the dose was unchanged. However, diarrhea scores decreased from 2.4 to 1.7 when the MMF dose was decreased. This effect of dose change was significant with a p-value of 0.02, see **Supplemental Figure S1** and **Table S3**.

### Troublesome Gastrointestinal Symptoms

Six months after kidney transplantation, 54 of the 72 recipients (75%) reported at least one troublesome gastrointestinal symptom (score ≥3 per question, **Figure 3**). Fifty percent of the recipients reported troublesome symptoms in more than one dimension (27 of the 52 recipients, 50%). Most reported troublesome symptoms were in the dimension indigestion (34 recipients), followed by abdominal pain (33 recipients), and diarrhea (31 recipients). Approximately half of the recipients had troublesome constipation symptoms (23 recipients) and fewer people reported troublesome reflux symptoms (12 recipients).

For recipients with troublesome complaints (at least one troublesome symptom per dimension) at month 6, the means were 3.3 for diarrhea, 2.8 for constipation, 2.8 for reflux, 2.7 for abdominal pain, and 2.7 for indigestion (**Table S4**).

Troublesome symptoms in TAC/MMF recipients developed as follows: Abdominal pain improved from 2.7 to 1.8 (M15), p=0.003; indigestion improved from 2.8 to 2.3 (M15), p=0.012; reflux improved from 2.9 to 1.7 (M15), p=0.007. Meanwhile, constipation did not improve (2.9 (M6) and 3.2 (M15), p=1.0), and diarrhea remained more or less stable (2.8 (M6) and 2.3 (M15), p=0.31).

### Effect of Discontinuing MMF on Troublesome Gastrointestinal Symptoms

The course of troublesome diarrhea differed in TACmono vs. TAC/MMF, with a decrease of 1.8 versus a decrease of 0.9, respectively, p= 0.03. The course of other symptoms did not differ between the groups (**Table S5**): for the dimension abdominal pain -0.6 vs. -0.9 (p=0.87), reflux -1.2 vs. -1.2 (p=0.96), indigestion -0.5 vs. -0.5 (p=0.95), and constipation -0.4 vs. +0.3 (p=0.39) in TACmono vs. TAC/MMF, respectively.

### Factors Influencing Diarrhea

As diarrhea was the most prominent finding, the effect of BMI, sex, age, and eGFR on diarrhea at M6, M12, and M15 was assessed. None of these variables correlated with diarrhea in uni- and multivariate analysis (**Supplemental Table S6**). Antibiotics were prescribed 16 times in 11 out of 72 recipients. Antibiotic use in the 3 months prior to either month 12 or month 15 was not related to any of the five gastrointestinal dimensions (**Supplemental Table S7**).

### Women Reported More Gastrointestinal Symptoms

Six months after kidney transplantation, women reported more gastrointestinal symptoms than men. Both abdominal pain (mean 1.9 vs. 1.6 in men, p=0.04) and constipation were more frequent in women (2.0 vs. 1.7 in men, p=0.03). Diarrhea tended to be more frequent in women (2.1 vs. 1.8 for men, p=0.09). These differences between men and women diminished over time and were no longer significant at month 15 (**Supplemental Table S8**). To study whether the effect of MMF on diarrhea was independent of sex, we performed a sensitivity analysis excluding women. The effect of discontinuing MMF on diarrhea over time remained significant in the analysis with male sex only (p= 0.04, data not shown).

## Discussion

This randomized controlled trial allowed for a systematic investigation of gastrointestinal symptoms in kidney transplant recipients with the most frequently used immunosuppressive regimen of tacrolimus (TAC) and mycophenolate mofetil (MMF). Six months after kidney transplantation, gastrointestinal symptoms were present in 90% of stable recipients. Troublesome symptoms were present in 75% of recipients (score of ≥3 out of 7). Indigestion, abdominal pain, and reflux diminished over time in recipients with troublesome symptoms. Discontinuing MMF led to a significant improvement in diarrhea. Unfortunately, constipation remained troublesome at 15 months after kidney transplantation in 42% of the recipients, irrespective of MMF use.

In the western world, gastrointestinal symptoms are present in 50%-60% of the general population (13, 14). This is a lower percentage than the 90% in our cohort of stable kidney transplant recipients. The question arises whether this burden of gastrointestinal symptoms as compared to the general population is caused by the kidney disease, by the immunosuppressive drugs, or by both. Gastrointestinal symptoms are common in end-stage renal disease, with a prevalence of 70% (15). Especially indigestion and reflux are common. In our study, indigestion and reflux were common at month 6, but diminished over time. A possible explanation is that these gastrointestinal symptoms had resulted from end-stage kidney disease, with gradual improvement after kidney transplantation. Abdominal pain, which was reported by 54% 6 months after transplantation, also diminished thereafter. This abdominal pain might result from the transplant surgery and its related complications (16). The decrease in time is in line with this explanation. Furthermore, TAC use has been associated with abdominal pain in past studies (17).

The high prevalence of gastrointestinal symptoms in our cohort is in line with transplantation literature, which reported a prevalence between 88%-92% (4, 17, 18). A similarity between these studies is the systematical assessment of gastrointestinal symptoms in stable kidney transplant recipients. A difference is that in our study, kidney transplant recipients were randomized to either continue or discontinue MMF. Corticosteroids had been discontinued in all recipients by month 5. The course of gastrointestinal symptoms could be studied by the longitudinal design of our study with three different time points.

Diarrhea is not a common complaint in end-stage kidney disease (15). In our study, diarrhea was the most troublesome complaint 6 months after kidney transplantation. This suggests that the immunosuppressive regimen could be a contributor to the diarrhea. This is supported by the finding that discontinuation of MMF led to a significant improvement of diarrhea. Adapting MMF dose based on therapeutic drug monitoring was related to a change in diarrhea complaints. Increasing the MMF dose based on lower range trough levels worsened diarrhea complaints and vice versa. The study of Chan et al. also reported the association between MMF and diarrhea (17). However, in their study, the use of MMF with TAC was compared to the use of other immunosuppressive drugs such as azathioprine, whereas in our study the use of TAC/MMF was compared to TAC monotherapy. Of note are two recipients who terminated our main study before randomization due to troublesome diarrhea, with complete resolution after discontinuation of MMF. After MMF discontinuation, recipients experienced less diarrhea complaints, but diarrhea was not completely resolved. These remaining complaints may be related to TAC use, as previous studies have shown an association between TAC and diarrhea and other gastrointestinal complaints (17).

Several possible mechanisms for diarrhea exist in kidney transplant recipients. It has been suggested that MMF causes diarrhea by causing more gastrointestinal infections due to its immunosuppressive effects (19). MMF has also been suggested to cause diarrhea due to inducing colitis, as diagnosed with intestinal biopsies (7, 20). The mechanism in which this process occurs is not completely understood. However, it is believed that enterocyte proliferation is affected by MMF, because MMF can block purine synthesis, which is essential for this process. It has also been suggested that MMF stimulates inflammatory processes in the gastrointestinal tract, *via* interleukin-6 and TNF-a release, which are pro-inflammatory cytokines (21). Furthermore, MMF has been linked to the formation of molecules that bind to the body’s cells, which can cause hypersensitivity and autoimmune reactions in the gastrointestinal tract (22). Studies have found that MMF-induced colitis can be histologically similar to a graft-versus-host reaction (23) or to inflammatory bowel disease (24–26). The influence of MMF on diarrhea is not a consistent finding in other reports, as Ekberg et al. and Ponticelli et al. found no association between MMF and diarrhea in kidney transplant recipients (4, 18). The daily dose of MMF in these studies was lower than in our study and had been changed if gastrointestinal symptoms were present.

Of note is that women experienced more gastrointestinal symptoms, in particular more abdominal pain and constipation. This finding is in line with Chan et al. (17) However, the reason is not clear. The menstrual cycle, menopause, and irritable bowel syndrome being more common in women and women having a lower threshold in reporting symptoms could play a role (27, 28).

The strength of our study is its randomized controlled design, in which MMF is not replaced by another immunosuppressive drug, but discontinued per protocol. To our knowledge this is the first RCT on gastrointestinal symptoms with this design, as past studies replaced MMF with enteric-coated MMF or other immunosuppressive drugs such as steroid of azathioprine (4, 17, 18, 29, 30).. It is also different from past studies where cyclosporine was often the backbone of the immunosuppressive regimen (4, 18, 25). Another strength in our study is that, despite our demographically mixed population, we did not exclude recipients with an indication of low literacy skills. Non-native speakers and people with low-literacy skills often remain outside the scope of clinical studies. Our research nurse assisted in filling in the questionnaires if needed, under the disclosure that the treating physician did not have access to the questionnaires.

Our study has several limitations. Only immunologically low-risk kidney transplant recipients were included, and randomization criteria such as kidney function were applied, resulting in a stable kidney transplant population without rejection episodes or failing allografts. Of course, this limits the extrapolation of findings to the general kidney transplant population. Because a lower kidney function is related to more gastrointestinal complaints, the incidence of gastrointestinal complaints in our study is a conservative estimate. The burden of gastrointestinal complaints in the general kidney transplant population is likely to be higher (31). Recipients filled in the questionnaires at 6- and 3-month intervals, so there may have been some recall bias.

Future research should focus on which factors contribute to the high prevalence of gastrointestinal symptoms in kidney transplant recipients. The rapidly evolving field of microbiota studies may help to unravel the mechanisms involved (32).

This is the first randomized controlled trial on gastrointestinal symptoms after kidney transplantation, in which MMF is not replaced by another immunosuppressive drug, but discontinued per protocol. The vast majority of kidney transplant recipients (90%) experienced gastrointestinal symptoms, and 75% reported troublesome symptoms. Constipation did not improve over time. Diarrhea was the most troublesome symptom and discontinuation of MMF significantly improved diarrhea symptoms.

## Data Availability Statement

The raw data supporting the conclusions of this article will be made available by the authors, without undue reservation.

## Ethics Statement

The studies involving human participants were reviewed and approved by Medical Ethical Committee Erasmus Medical Center. The patients/participants provided their written informed consent to participate in this study.

## Author Contributions

ZA is the first author who has done most of the analysis and written most of the manuscript. MB is a co-author who has helped substantially with interpreting the data, the data analysis and writing the manuscript. JG and MV are researchers who have helped with collecting the data and providing it in the right form and critically revising the manuscript. AW is the supervisor who has supervised all the work from the beginning and helped with interpreting the data, with the analysis, and with writing the manuscript. All authors have helped with revising the manuscript critically and they all provide approval for publication of the content and agree to be accountable for all aspects of the work in ensuring that questions related to the accuracy or integrity of any part of the work are appropriately investigated and resolved.

## Conflict of Interest

The authors declare that the research was conducted in the absence of any commercial or financial relationships that could be construed as a potential conflict of interest.

## Publisher’s Note

All claims expressed in this article are solely those of the authors and do not necessarily represent those of their affiliated organizations, or those of the publisher, the editors and the reviewers. Any product that may be evaluated in this article, or claim that may be made by its manufacturer, is not guaranteed or endorsed by the publisher.

## Supplementary Material

Supplementary Figure 1 Effect of Mycophenolate mofetil dose adaptation on mean diarrhea scores between month 6 and month 12 after kidney transplantation.
