# Supplementary figures and images for: The Burden of Gastrointestinal Complaints in Kidney Transplant Recipients Using Tacrolimus With and Without Mycophenolate Mofetil: A Randomized Controlled Study
Source: Front Nephrol. 2022 Jul 19;2:933954. doi: 10.3389/fneph.2022.933954 (PMC10479617; doi:10.3389/fneph.2022.933954)

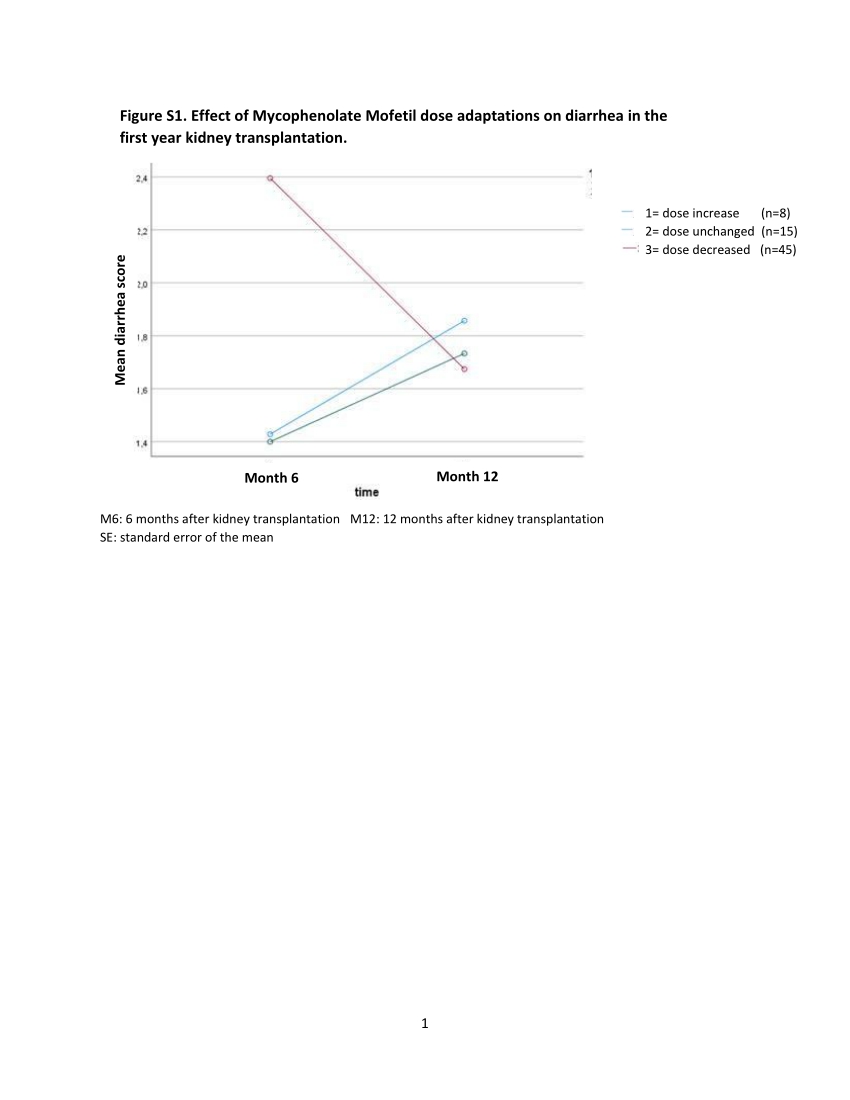

Supplement: Supplementary Figure 1 — Effect of Mycophenolate mofetil dose adaptation on mean diarrhea scores between month 6 and month 12 after kidney transplantation. [file Image_1.jpeg]
