# Supplementary material for: The Burden of Gastrointestinal Complaints in Kidney Transplant Recipients Using Tacrolimus With and Without Mycophenolate Mofetil: A Randomized Controlled Study
Source: Front Nephrol. 2022 Jul 19;2:933954. doi: 10.3389/fneph.2022.933954 (PMC10479617; doi:10.3389/fneph.2022.933954)
Supplement: Supplementary file 2 [file DataSheet_1.pdf]

Supplemental material

Table S1. Inclusion, exclusion and randomization criteria of Tacrolimus monotherapy in immunologically low-risk kidney transplant recipients: a randomized-controlled trial. Netherlands Trial Register [NTR4672, www.trialregister.nl]

**transplantation**	**inclusion**	aged ≥18 years	**exclusion**	ABO-incompatibility
		peakPRA <5%		CDC or FACS positive crossmatch
		HLA mismatch <4		
		absence of immunological renal disease		multi-organ transplantation
				females of childbearing potential unwilling to use effective means of contraception
**month 6**	**Randomization**	eGFR >30 ml/min		biopsy-proven rejection after 3 months
		proteinuria ≤ 50 mg/mmol in spot urine		lymphocyte depleting therapy

PRA: Panel-reactive antibody CDC: Complemet-dependent cytotoxicity FACS: fluorecense activated cell sorting
HLA: human leukocyte antigen eGFR: estimated gromerular filtration rate

Table S2. Tacrolimus and mycophenolate mofetil trough levels in kidney transplant recipients using tacrolimus with and without mycophenolate mofetil 6, 9, 12 and 15 months after transplantation.							
	**TACmono**			**TAC/MMF**			***p-value***
**N**	34			38			
	Median in ug/L (TAC) or mg/L (MMF) (IQR)	Below range %	Above range %	Median in ug/L (TAC) or mg/L (MMF) (IQR)	Below range %	Above range %	(of levels outside target level)
**Tacrolimus**							
M6	7.4 (2.8)	9	44	7.2 (2.8)	24	29	0.98
M9	6.3 (1.8)	15	18	5.9 (1.9)	18	5	0.29
M12	6.1 (1.8)	18	12	6.2 (1.3)	18	8	0.44
M15	6.3 (1.1)	18	6	6.1 (1.6)	16	11	0.79
**Mycophenolate mofetil**							
M6	2.2 (1.5)	35	15	1.8 (1.4)	29	18	0.83
M9	-	-	-	1.5 (1.4)	32	13	-
M12	-	-	-	2.0 (2.1)	29	32	-
M15	-	-	-	1.9 (2.0)	32	24	-

N: number TAC: tacrolimus MMF: mycophenolate mofetil IQR: interquartile range
M6: 6 months after transplantation M9: 9 months after transplantation M12: 12 months after transplantation
M15: 15 months after transplantation.

**Table S3. Effect of Mycophenolate Mofetil dose adaptations on diarrhea in the first year after kidney transplantation.**						
**Adaptation**	**n**	**Time**	**Mean diarrhea score**	**SE**	**95% Confidence Interval**	
					**Lower Bound**	**Upper Bound**
**Increased dose**	8	M6	1.4	0.5	0.40	2.46
		M12	1.9	0.4	1.06	2.66
**Unchanged dose**	15	M6	1.4	04	0.70	2.11
		M12	1.7	0.3	1.19	2.28
**Decreased dose**	45	M6	2.4	0.2	1.98	2.81
		M12	1.7	0.2	1.35	1.20

M6: 6 months after kidney transplantation   M12: 12 months after kidney transplantation
SE: standard error of the mean

**Table S4. Upper and lower gastrointestinal symptoms in kidney transplant recipients treated with tacrolimus with mycophenolate mofetil versus tacrolimus without mycophenolate mofetil.**				
**Time**	**Category**	**TACmono**	**TAC/MMF**	***p-value***
**Month 6**	Upper	1.64 (0.81)	1.65 (0.86)	0.96
	Lower	1.64 (0.81)	1.65 (0.86)	0.96
**Month 12**	Upper	1.56 (0.75)	1.44 (0.60)	0.44
	Lower	1.68 (0.95)	1.82 (0.85)	0.50
**Month 15**	Upper	1.52 (0.80)	1.62 (0.71)	0.57
	Lower	1.76 (0.87)	1.97 (1.01)	0.34

*Repeated measures analysis

Mean scores and standard deviations are reported per upper and lower category.
TACmono: tacrolimus monotherapy   TAC: tacrolimus   MMF: mycophenolate mofetil

**Table S5. The course of severe gastrointestinal symptoms in kidney transplant recipients from 6 months onwards per treatment group.**				
**Time**	**Dimension**	**Overall mean (SD)**	**TAC-mono mean (SD)**	**TAC/MMF mean (SD)**
**Month 6**	**Abdominal pain n= 33**	2.7 (0.88)	2.7 (1.01)	2.7 (0.81)
	**Reflux n=12**	2.8 (1.01)	2.6 (0.55)	2.9 (1.35)
	**Indigestion n= 34**	2.7 (1.04)	2.6 (1.01)	2.8 (1.10)
	**Constipation n=23**	2.8 (1.10)	2.6 (1.03)	2.9 (1.17)
	**Diarrhea n=31**	3.3 (1.30)	3.8 (1.52)	2.8 (0.83)
**Month 12**	**Abdominal pain**		2.0 (1.00)	1.8 (1.02)
	**Reflux**		1.2 (0.45)	1.9 (0.90)
	**Indigestion**		2.0 (0.88)	2.1 (0.95)
	**Constipation**		2.4 (1.12)	2.7 (1.60)
	**Diarrhea**		1.7 (0.96)	2.3 (1.30)
**Month 15**	**Abdominal pain**		2.1 (1.12)	1.8 (0.90)
	**Reflux**		1.4 (0.89)	1.7 (1.11)
	**Indigestion**		2.1 (1.03)	2.3 (0.83)
	**Constipation**		2.2 (1.17)	3.2 (1.64)
	**Diarrhea**		2.0 (1.13)	2.3 (1.29)
**Change over time from 6 till 15 months after transplantation.**				
		**Δ score**	**Δ score**	***p-value***[#]
	**Abdominal pain**	-0.6	-0.9	0.87
	**Reflux**	-1.2	-1.2	0.96
	**Indigestion**	-0.5	-0.5	0.95
	**Constipation**	-0.4	+0.3	0.39
	**Diarrhea**	-1.8	-0.5	0.03

*Repeated measures analysis

TACmono: tacrolimus monotherapy TAC: tacrolimus MMF: mycophenolate mofetil SD: standard deviation

N: number Δ: change

Table S6. Diarrhea multivariate analysis			
**Time**	**Variabele**	**Correlation**	***p-value***
**Month 6**	BMI	0.043	0.98
	Sex	0.385	0.71
	Age	0.015	0.50
	eGFR	0.011	0.66
	Proteinuria	0.022	0.77
**Month 12**	BMI	0.03	0.53
	Sex	0.277	0.27
	eGFR	0.007	0.14
	Proteinuria	0.010	0.22
**Month 15**	BMI	0.03	0.66
	Sex	0.30	0.11
	eGFR	0.008	0.69
	Proteinuria	0.009	0.47

BMI: body mass index eGFR: estimated glomerular filtration rate

Table S7. Gastrointestinal complaints in kidney transplant recipients with and without recent antibiotic use.			
	**With recent antibiotic use (n=16)**	**Without recent antibiotic use (n=128)**	***p-value***
**Abdominal pain**	1.6	1.6	0.62
**Reflux**	1.3	1.3	0.68
**Indigestion**	1.5	1.8	0.12
**Constipation**	2.1	1.8	0.44
**Diarrhea**	1.9	1.7	0.49

N: number

**Table S8. Gastrointestinal symptoms in men versus women six months after kidney transplantation.**			
**Dimension**	**Sex**	**Mean**	***p-value*** ***Bonferroni correction: significance level <0.01***
**Abdominal pain**	Male	1.56	0.04
	Female	1.87	
**Indigestion**	Male	1.78	0.40
	Female	1.90	
**Reflux**	Male	1.31	0.28
	Female	1.45	
**Constipation**	Male	1.66	0.03
	Female	2.03	
**Diarrhea**	Male	1.77	0.09
	Female	2.08	
